# Flavonoids Alleviate Peripheral Neuropathy Induced by Anticancer Drugs

**DOI:** 10.3390/cancers13071576

**Published:** 2021-03-29

**Authors:** Manaal Siddiqui, Basma Abdellatif, Kevin Zhai, Alena Liskova, Peter Kubatka, Dietrich Büsselberg

**Affiliations:** 1 Weill Cornell Medicine-Qatar, Education City, Qatar Foundation, Doha 24144, Qatar; mas4002@qatar-med.cornell.edu (M.S.); bwa4001@qatar-med.cornell.edu (B.A.); kez4003@qatar-med.cornell.edu (K.Z.); 2 Department of Obstetrics and Gynecology, Jessenius Faculty of Medicine, Comenius University in Bratislava, 036 01 Martin, Slovakia; liskova80@uniba.sk; 3 Department of Medical Biology, Jessenius Faculty of Medicine, Comenius University in Bratislava, 036 01 Martin, Slovakia; peter.kubatka@uniba.sk

**Keywords:** flavonoids, CIPN, chemotherapy, peripheral neuropathy

## Abstract

**Simple Summary:**

Chemotherapy-induced peripheral neuropathy (CIPN) is a debilitating condition that severely reduces the quality of life of a considerable proportion of cancer patients. There is no cure for CIPN to date. Here, we explore the potential of flavonoids as pharmacological agents in combating CIPN. Flavonoids alleviate CIPN by reducing oxidative stress, inflammation, and neuronal damage, among other mechanisms. Future research should evaluate the efficacy and side effects of flavonoids in human models of CIPN.

**Abstract:**

Purpose: This study aimed to assess the potential of flavonoids in combating CIPN. Methods: PubMed and Google Scholar were used, and studies that investigated flavonoids in models of CIPN and models of neuropathic pain similar to CIPN were included. Only studies investigating peripheral mechanisms of CIPN were used. Results: Flavonoids inhibit several essential mechanisms of CIPN, such as proinflammatory cytokine release, astrocyte and microglial activation, oxidative stress, neuronal damage and apoptosis, mitochondrial damage, ectopic discharge, and ion channel activation. They decreased the severity of certain CIPN symptoms, such as thermal hyperalgesia and mechanical, tactile, and cold allodynia. Conclusions: Flavonoids hold immense promise in treating CIPN; thus, future research should investigate their effects in humans. Specifically, precise pharmacological mechanisms and side effects need to be elucidated in human models before clinical benefits can be achieved.

## 1. The Burden of CIPN and the Hope of Flavonoids

Chemotherapy-induced peripheral neuropathy (CIPN) is a common side effect of cancer treatment with antineoplastic agents. The neurotoxic effects of anticancer drugs lead to neuropathy in about 70% (up to 90% for oxaliplatin) of cancer patients [1]. Anticancer drugs that commonly induce peripheral neuropathy include plant alkaloids (e.g., vincristine, vinorelbine, and vinblastine), taxanes (e.g., docetaxel, paclitaxel, and cabazitaxel), platinum drugs (e.g., cisplatin and carboplatin), immunomodulatory drugs (e.g. thalidomide and pomalidomide), and proteasome inhibitors (e.g., bortezomib) [2]. These drugs damage sensory, motor, and autonomic nerves through various mechanisms, resulting in neuronal degradation [1]. Existing evidence shows that these mechanisms include proinflammatory cytokine release, oxidative stress, mitochondrial damage, ion channel activation, ectopic discharge, microglial enhancement, and astrocyte activation. Besides these, other neuroinflammatory mechanisms are involved in the pathogenicity of CIPN [3,4,5,6,7]. Furthermore, there is evidence of a unique pattern of central pain processing in patients with CIPN; for example, the precuneus, a region implicated in conscious pain perception, is significantly more active in people with CIPN than in normal individuals during painful stimulation. Patients with CIPN also exhibit reduced activation of the right superior frontal gyrus, an area associated with subjectively unpleasant experiences such as pain [8].

CIPN can be acute, starting days or hours after chemotherapy, or chronic, starting several months after chemotherapy [7,9]. CIPN symptoms tend to worsen over time; this phenomenon is known as the “coasting effect” [10]. Symptoms include tingling, burning, freezing, electroshock-like sensations, numbness, weakness, and pain [2,10]. These symptoms have symmetrical effects and often follow a stocking and glove distribution, affecting the extremities [1]. CIPN is a debilitating condition that interferes with daily activities, causing emotional distress and reducing the quality of life [11,12]. Since its symptoms are dose-dependent, CIPN is also the leading cause of premature chemotherapy cessation, leading to reduced life span [1,10]. Moreover, peripheral neuropathy itself is associated with all-cause mortality in adults and can therefore be used as a marker for death risk [13].

Some treatments employed to manage CIPN include steroids, antidepressants such as duloxetine, numbing medications such as lidocaine and capsaicin, and antiseizure medications such as gabapentin and pregabalin [14,15]. While opioids are prescribed for those with extraordinarily severe or chronic CIPN, this practice is discouraged by the Centers for Disease Control due to the opioid epidemic [15]. Current treatments are based on other neuropathic pain models (diabetic, herpetic, and others) and have limited efficacy in CIPN.

Furthermore, there is no evidence for the pharmacological efficacy of antiseizure and antidepressant medications in treating CIPN [15]. Duloxetine is the only nonopioid pharmacological medication with consistent efficacy [15]. Unfortunately, recent in vivo and in vitro studies on CIPN states have yielded no major pharmacological advancements. Moreover, the majority of patients with CIPN receive no medical treatment for their symptoms [16]. These factors underscore the need for standardized, reliable treatments to improve cancer patients’ quality of life. Prior studies of CIPN mechanisms need to be thoroughly re-examined in light of contemporary studies investigating treatments. One class of compounds with the potential to relieve CIPN is flavonoids.

Flavonoids are secondary plant metabolites ubiquitous in fruits, vegetables, flowers, and barks [17]. They are phenolic compounds with variously substituted three-ring structures [18]. Flavonoids exert anti-inflammatory, antioxidant, anxiolytic, neuroprotective, and anti-nociceptive properties [17], which grant alleviative effects in neuropathic pain models such as CIPN, diabetic neuropathy, and sciatic nerve chronic constriction injury [18,19]. Flavonoids’ anticancer properties depend on the previously mentioned activities and their apoptotic, anti-angiogenic effects; they also target the Warburg effect [20,21,22]. The neuroprotective effects of flavonoids may stem from their interference with the serotonin, dopamine, GABA, and glycine neurotransmitter (NT) pathways [18].

**Figure 1:**
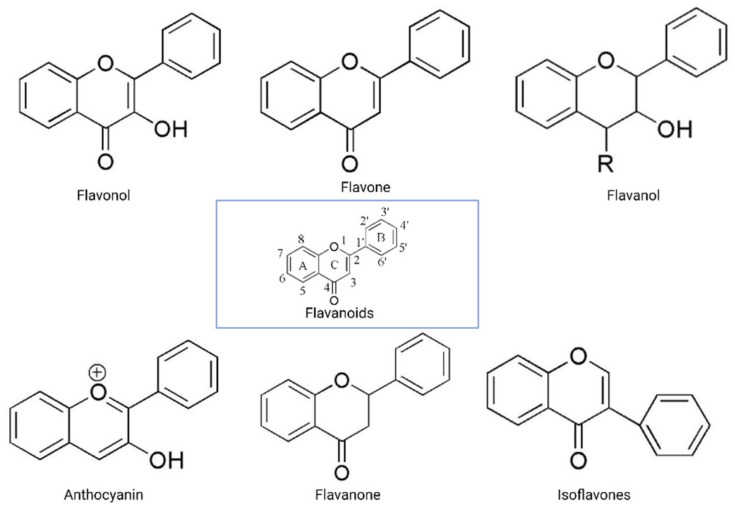
General structure of flavonoids. Adapted from Wang et.al. [24].

The flavonoid backbone consists of 15 carbon atoms arranged in the form of C6-C3-C6. Thus, it includes two benzene rings (A and B) connected by three carbon atoms, which may form a third (C) ring [17,23]. Structurally effective flavonoids consist of a catechol substructure on the A or B ring, a C3-OH group on the C ring, and an oxo group on C4. Above mentioned flavonoids’ beneficial effects are enhanced by a double bond between C2 and C3 due to planar molecule formation and increased double bond conjugation Figure 1 [23]. According to the carbon of the C ring, which is connected to the B ring and the oxidation and degrees of unsaturation of the C ring, flavonoids are classified into the following sub-groups: anthocyanins, flavanones, flavones, flavonols, flavanols, and isoflavones [17,23].

Structurally, flavones have two benzene rings linked by a heterocyclic pyrone ring (2-phenyl-chromones) [25]; essential members of this class include luteolin, apigenin, isoorientin, and icariin. Flavanones are dihydroflavones; therefore, the C ring and the bond between positions 2 and 3 are saturated [26]. Examples of flavanones are hesperetin, naringenin, silibinin, and eriodictyol. Flavanols–which include catechins and epicatechin–are the 3-hydroxy derivatives of flavanones [26]. Isoflavones have estrogenic properties and structures that include 3-phenyl-benzopyrone [27]; the main isoflavones are genistein, daidzein, biochanin A, and glycitein. Anthocyanins’ basic structural unit is the flavylium cation (2-phenylbenzopyrilium); this confers a positive charge on the oxygen skeleton. Most anthocyanins are acylated by organic acids through ester bonds. Important group members include cyanidin, delphinidin, malvidin, and peonidin. Finally, flavonols, such as quercetin, morin, and kaempferol, have a 2-phenyl-3-hydroxy- chromone backbone; rutin is a flavonol glycoside composed of quercetin and disaccharide rutinose [28,29]. The chemical structures of flavonoid classes can be seen in Figure 1.

**Figure 2:**
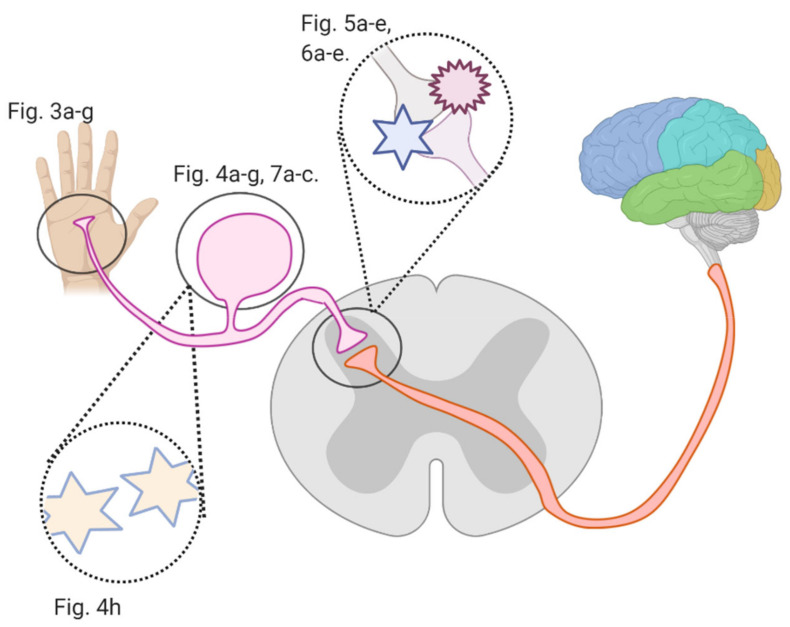
An overview of this article’s figures illustrating Regions where flavonoids interfere with signal transmission in CIPN; Figure 3a–g describe flavonoids’ and anticancer drugs’ actions at the periphery. Figure 4a–f describe flavonoids’ and anticancer drugs’ actions at the dorsal root ganglion. Figure 5a–d describe flavonoids’ and anticancer drugs’ actions at the spinal dorsal horn. Figure 6a–d describe flavonoids’ and anticancer drugs’ actions in astrocytes and glial cells. Figure 7a–b describe flavonoids’ and anticancer drugs’ roles in neuronal injury.

Several studies investigated flavonoids’ role in counteracting CIPN and reversing related oxidative stress and neuronal damage. The growing burden of CIPN and the emerging potential of flavonoids necessitate an analysis of the mechanisms by which flavonoids counter CIPN. Here, we review these mechanisms (see Figure 2) in the periphery, dorsal root ganglion, and spinal cord dorsal horn synapse, as well as in astrocytes and microglial cells.

## 2. Materials and Methods

PubMed and Google Scholar were searched using the following keywords: “flavonoids”, “CIPN”, “neuropathic pain”, and “peripheral neuropathy”. One hundred thirty-four results were obtained for the combination “flavonoids” AND “CIPN”, 6620 for “flavonoids” AND “neuropathic pain”, and 4909 for “flavonoids” AND “peripheral neuropathy”. We included studies that investigated the effects of flavonoids on models of CIPN, sciatic nerve chronic constriction injury (CCI), partial sciatic nerve ligation (PNL), spared nerve injury (SNI), and spinal nerve ligation (SNL); the latter four share mechanisms with CIPN. We included studies with nerve injury models only if their findings were likely to be generalizable to CIPN (i.e., they investigated a mechanism in common with CIPN). Studies on diabetic, herpetic, or other clinically manifesting peripheral neuropathies apart from CIPN were excluded. We only included studies discussing peripheral nervous system mechanisms. These inclusion criteria encompassed 8 studies on CIPN and flavonoids; we selected seven. Similarly, we found 18 studies investigating flavonoids with CCI, SNL, PNL, and SNI, and included four. 

## 3. Flavonoids Counter the Effects of Anticancer Drugs at the Peripheral Nociceptor

### 3.1. General Effects of Anticancer Drugs and Flavonoids

**Figure 3:**
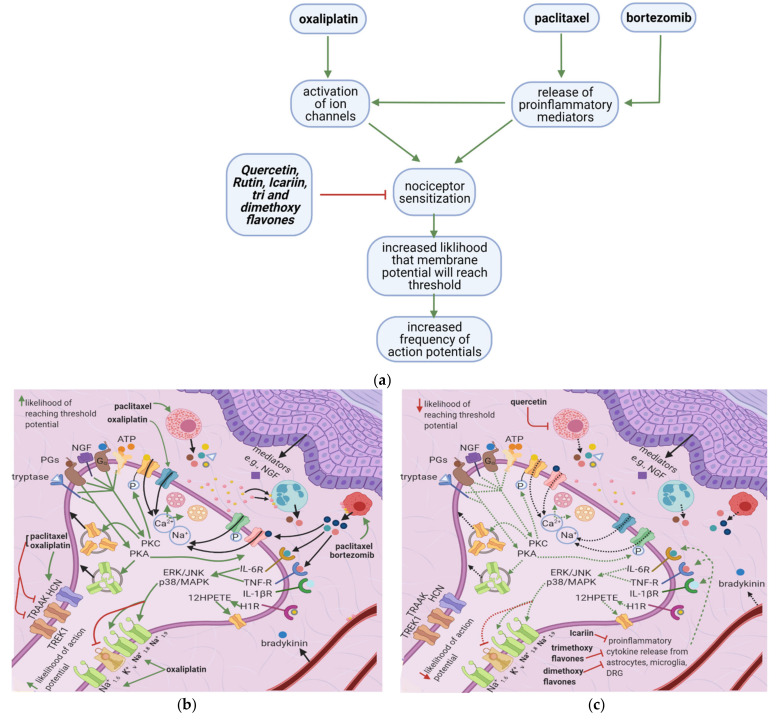

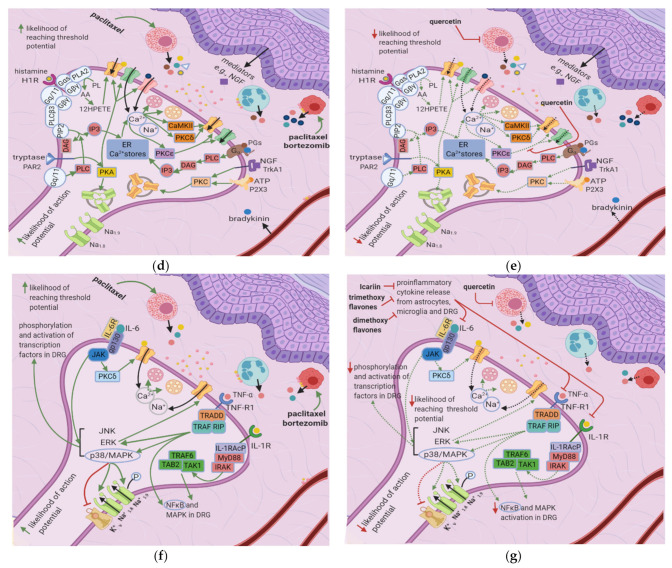
Effects of anticancer drugs and flavonoids at the peripheral nociceptor. (**a**) Simplified Overview. Flavonoids counter anticancer drug induced nociceptor sensitization. Anticancer drugs, via the enhanced release of proinflammatory cytokines and activation of ion channels, sensitize the nociceptor, increasing the likelihood that the membrane potential will reach the threshold potential. Thus, there is a greater chance that action potentials will result from weak stimuli that under normal conditions would not reach the threshold potential. (**b**) Effects of anticancer drugs on the peripheral nociceptor. Anticancer drugs increase the release of proinflammatory mediators such as TNF-α, IL-1β, IL-6, and histamine. These mediators directly sensitize ion channels and activate the ERK/JNK and p38/MAPK pathways, resulting in the activation of Na^+1.8^ and Na^+1.9^ and the inhibition of K^+v^. Histamine, prostaglandins, and tryptase bind to their respective receptors and activate the PKA and PKC pathways, which increase the membrane density of TRPV1 and Na^+^_v_ channels. These events ultimately elevate the membrane potential to the threshold value, increasing the likelihood of an action potential; (**c**) Actions of flavonoids counter anticancer drugs’ effects. Icariin and trimethoxy and dimethoxy flavones decrease the release of TNF-α, IL-1β, and IL-6 from astrocytes, microglia, and the DRG, and thereby downregulate the ERK/JNK and p38/MAPK pathways. This reduces the membrane density of ion channels, and consequently decreases the likelihood of reaching the threshold potential and reduces pain signal transmission; (**d**) Activation of PKA and PKC derivatives by anticancer drugs. Paclitaxel induces the degranulation of mast cells, which release tryptase and other proinflammatory mediators. Tryptase acts on PAR2, leading to PKA activation, the sensitization of TRPV1, TRPA1, and TRPV4, and the increased membrane fusion of Na^+^_v_. The sensitization of TRP channels increases Na^+^ and Ca^2+^ inflow into the nociceptor. Ca^2+^ causes vesicles to fuse with the membrane and activates PKCδ, CaMKII, and PKC∈, which activate TRPV4 and TRPV1, further increasing ion inflow. These events increase the likelihood that the membrane potential will reach the threshold potential; (**e**) Flavonoids prevent neuropathic pain, affecting PKA and PKC derivatives activation. Quercetin inhibits the translocation of PKC∈ from the cytoplasm to the membrane and prevents paclitaxel-induced mast cell degranulation. Thus, there is less activation of PKA and PKC derivatives, which leads to decreased channel activation, less ionic influx, and a reduced likelihood that the membrane potential will reach threshold potential; (**f**) Anticancer drugs lead to the release of proinflammatory cytokines, which sensitize the nociceptor. Proinflammatory cytokines act directly on and sensitize TRP channels. They also increase the phosphorylation of transcription factors in the DRG via the p38/MAPK, ERK, and JNK pathways, increasing the synthesis of primary afferent channels; (**g**) Flavonoids (icariin and trimethoxy and dimethoxy flavones) reduce the release of proinflammatory cytokines by astrocytes, microglia, and the DRG. Thus, they downregulate the p38/MAPK, ERK, and JNK pathways, decrease ion channel density and phosphorylation, and consequently decrease the likelihood that the membrane potential will reach the threshold potential.

Oxaliplatin, paclitaxel, and bortezomib destabilize the nociceptor membrane by elevating the resting membrane potential toward the threshold; this increases the likelihood of an action potential. Oxaliplatin activates ion channels involved in action potential initiation and propagation [10,16]. Paclitaxel and bortezomib enhance the release of proinflammatory cytokines, which sensitize peripheral nociceptors [1,2,19] (Figure 3a). Icariin, trimethoxyflavones, and dimethoxyflavones decrease proinflammatory cytokine release and reduce mechanical allodynia [13,14,15]. Moreover, quercetin inhibits paclitaxel-induced mast cell degranulation, reducing the release of inflammatory mediators and decreasing the thermal hyperalgesia and mechanical allodynia thresholds [12].

### 3.2. Effects of Anticancer Drugs

#### 3.2.1. Ion Channel Activation

Oxaliplatin upregulates the expression of transient receptor potential melastatin 8 (TRPM8) [10,16], causing cold allodynia. It also activates TTX-R Na^+^_1.8_ (involved in action potential initiation), TTX-R Na^+1.6^, and HCN (hyperpolarization-activated channels involved in action potential propagation) while inhibiting TREK1 and TRAAK (potassium channels which restore the membrane potential to the resting state) [11]. Furthermore, nerve growth factor (NGF) and ATP activate protein kinase C (PKC) through their actions on TrkA1 and P2X3, respectively; PKC, in turn, increases the membrane fusion of transient receptor potential vanilloid 1 (TRPV1) vesicles [17,18]. These increases in ion channel activity and density destabilize the nociceptor membrane and cause the neuron to exhibit oscillatory behavior.

#### 3.2.2. Release of Proinflammatory Mediators

Paclitaxel induces mast cell degranulation [19], causing the release of the inflammatory mediators TNF-∝, IL-1β, IL-6, histamine, prostaglandins, and tryptase [20]. Paclitaxel and bortezomib activate macrophages [1,2], which release ROS, TNF-∝, and IL-1β [21]. These mediators act on their respective receptors, inducing a series of events that raise the membrane potential [22] (Figure 3b).

ROS directly acts on and sensitizes transient receptor potential ankyrin 1 (TRPA1) [23] (Table 1), while TNF-∝ and IL-1β act on TNF-R and IL-1R, respectively, leading to extracellular signal-regulated kinase (ERK) activation and p38/MAPK signaling [17] (Figure 3d). IL-1β also acts directly on TRPV1, increasing ionic inflow through this channel [17]. IL-6 stimulates IL-6R, causing the activation of c-Jun N-terminal kinases (JNK), ERK, and p38/MAPK [17]. The IL-6/IL6-R complex subsequently activates PKCδ, which sensitizes TRPV1 [24]. Activation of p38/MAPK leads to phosphorylation of Na^+1.8^ and Na^+1.9^ and inhibition of K^+v^ activity [8,17,25]. The recruitment of ERK, JNK, and p38/MAPK pathways result in increased phosphorylation and activation of transcription factors in the DRG; thus, more ion channels are synthesized (long-term changes) [17] (Figure 3f). Changes in ion channel density on the nociceptor membrane raise the resting membrane potential toward the threshold, increasing the likelihood that an action potential will result from stimuli too weak to cause one under normal conditions.

Histamine, tryptase, prostaglandins, and bradykinin cause a cascade of events that ultimately increase intracellular IP3 levels [6,18,20]. IP3 causes Ca^2+^ release from intracellular Ca^2+^-stores; increased intracellular Ca^2+^ increases the membrane fusion of vesicles containing substance P and cGRP [18]. These neuropeptides act on polymorphonuclear leukocytes, macrophages, and blood vessels, causing them to release increased amounts of proinflammatory mediators in a positive feedback loop [18]. Intracellular Ca^2+^ also activates PKCδ and CaMKII, which sensitizes TRPV1 [18], and PKC∈, which sensitizes TRPV4 and TRPV1 [6] (Figure 3d). The tryptase/PAR2 complex also leads to PKA activation, which causes the membrane fusion of vesicles containing TTX-R Na^+^-channels and sensitizes the TRPV1, TRPA1, and TRPV4 channels [6,17]. Furthermore, the histamine/H1R complex leads to the activation of PLA2, which increases the conversion of phospholipase to arachidonic acid. Arachidonic acid is subsequently converted to 12-HPETE, which sensitizes TRPV1 [26,27].

### 3.3. Flavonoids Counteract the Effects of Anticancer Drugs

#### 3.3.1. Icariin, Trimethoxy- and Dimethoxyflavones

In paclitaxel-induced models of CIPN, the flavonoid icariin inhibited the release of IL-1β, TNF-∝, and IL-6 from the DRG, astrocytes, and microglia [13], while trimethoxy and dimethoxy flavones inhibited the release of IL-1β, TNF-∝, and free radicals [14,15] (Table 2). Decreased action of proinflammatory cytokines on the nociceptor would render p38/MAPK, ERK, and JNK less active, decreasing the activation and synthesis of TTX-Resistant Na^+^_v_ channels (Figure 3g). Thus, the membrane potential would be less likely to reach the threshold potential (Figure 3c). These factors may account for the reduction of tactile allodynia and thermal hyperalgesia by trimethoxy and dimethoxy flavones and of mechanical allodynia by icariin [13,14,15].

#### 3.3.2. Quercetin

In a paclitaxel-induced model of CIPN, quercetin inhibited the degranulation of mast cells and thereby reduced histamine release [12]. Decreased histamine-HIR pathway activation reduced HPETE release, IP3 activation, TRPV1 activation, and intracellular Ca^2+^ release. Quercetin also inhibits PKC epsilon movement from the cytoplasm to the membrane [12]; thus, there is lesser activation of TRPV1 and TRPV4 [6]. Overall, quercetin decreases the intraneuronal concentrations of Ca^2+^ and Na^+^, decreasing the likelihood that the membrane potential will reach the threshold potential. This explains the decrease in thermal hyperalgesia and mechanical allodynia thresholds observed by Gao et al. [12]. Mechanisms of CIPN at the peripheral nociceptor induced by anticancer therapy and the role of flavonoids that counter these effects are summarized in Table 1.

## 4. Flavonoids Counter the Effects of Anticancer Drugs at the Dorsal Root Ganglion

### 4.1. General Effects of Anticancer Drugs and Flavonoids

**Figure 4:**
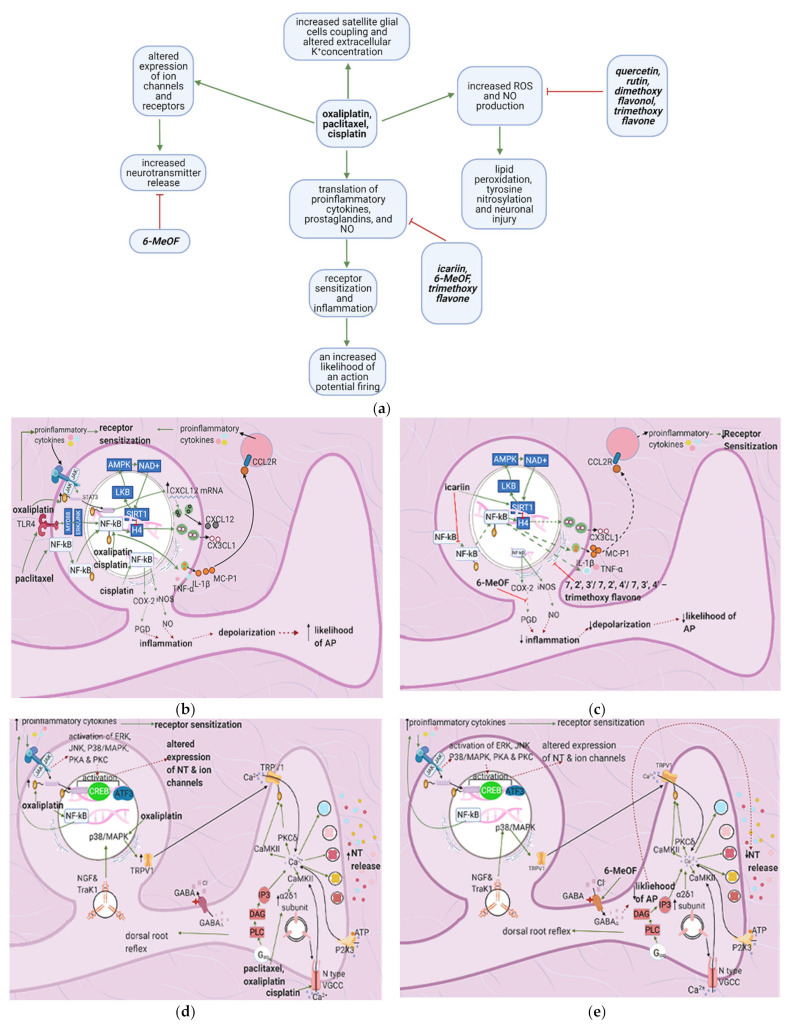

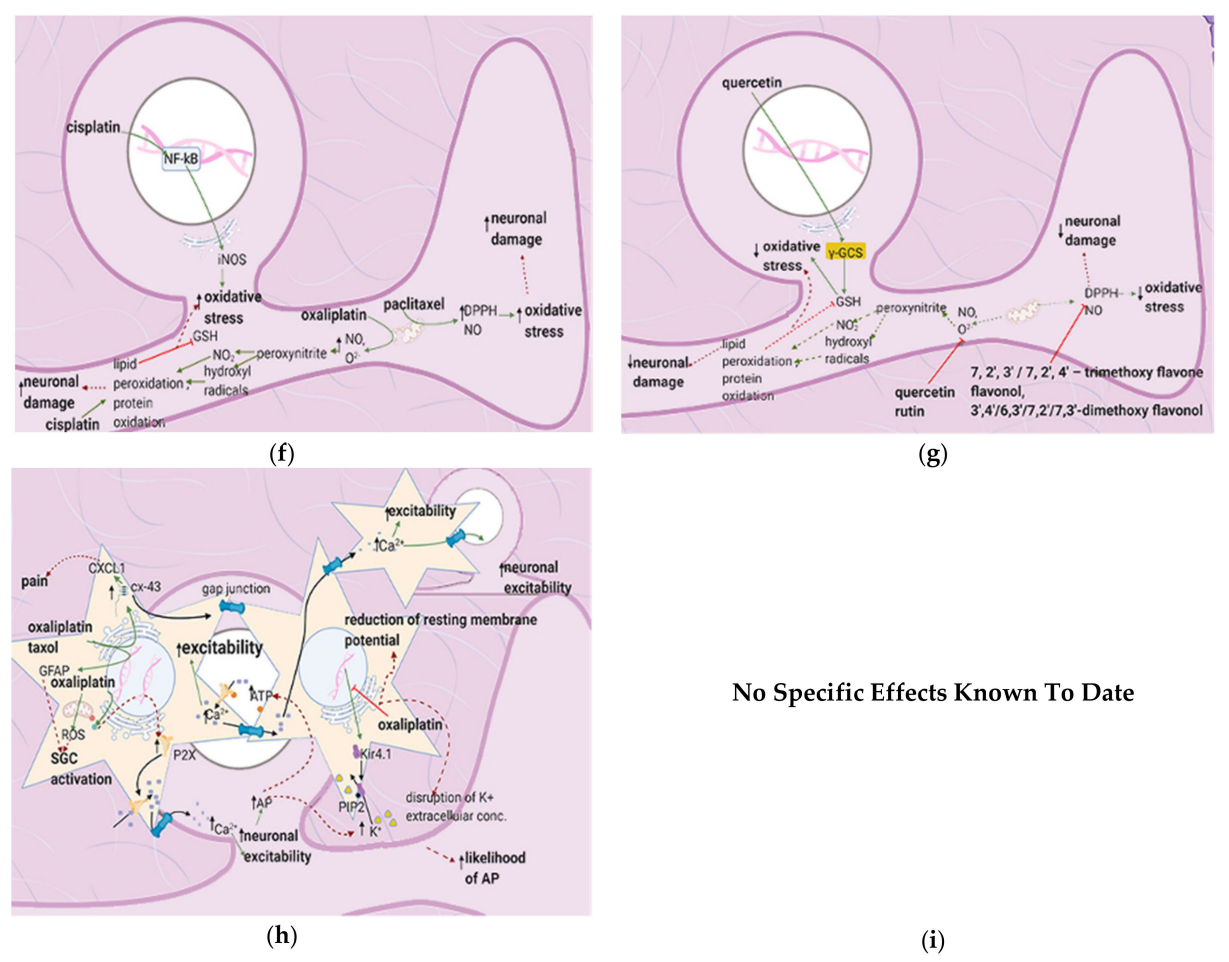
The actions of anticancer drugs (left column) and flavonoids (right column) on a dorsal root ganglion. (**a**) Simplified Overview. Flavonoids counteract anticancer drugs and increase mitochondrial damage, proinflammatory cytokine production, receptor sensitization, and the likelihood of an action potential. Also depicted is how anticancer drugs increase satellite glial cell coupling and excitability. (**b**) Anticancer Drugs on Proinflammatory Cytokine Production at the DRG. Anticancer drugs increase the translation of proinflammatory cytokines, prostaglandins, and NO through SIRT1 pathway downregulation and NF-κB activation. This leads to receptor sensitization and an increased likelihood of an action potential firing; (**c**) Flavonoids on Anticancer Drug- Induced Proinflammatory Cytokine Production in the DRG. Flavonoids counteract anticancer drugs’ actions at the DRG by inhibiting the translocation of NF-κB, upregulating the SIRT1 pathway, and inhibiting the production of proinflammatory cytokines and prostaglandins. These actions decrease inflammation and consequently decrease the likelihood of AP firing and receptor sensitization; (**d**) Anticancer Drugs on Ion Channels and NT Production at the DRG. Anticancer drugs increase the expression of ion channels such as TRPV1 and N-type VGCC, increasing intracellular Ca^2+^ concentrations and consequently increasing NT release. It also results in a dorsal root reflex; (**e**) Flavonoids on Anticancer Drug-Induced Increases in NT Production and Ion Channel Expression. 6-MeOF triggers GABAA receptors, decreasing an AP’s likelihood and consequently reducing NT release and alleviating pain; (**f**) Anticancer Drugs on the DRG’s Oxidative Stress Level. Anticancer drugs increase mitochondrial oxidant production, resulting in lipid peroxidation and tyrosine nitrosylation, lowering GSH levels in the cell and increases oxidative stress, causing neuronal damage; (**g**) Flavonoids on Anticancer Drug-Induced Increases in DRG Oxidative Stress. Flavonoids counteract the increase in oxidative stress by increasing the translation of GSH and directly scavenging reactive oxygen species; (**h**) Anticancer Drugs on Satellite Glial Cells. Anticancer drugs increase the expression of gap junctions that connect astrocytes surrounding the same and different DRGs. They also connect SGCs and neurons. Ca^2+^ waves travel through these gap junctions, reducing the membrane potential of SGCs and increasing the likelihood of AP firing in neurons. The Ca^2+^ waves are hypothetically generated by the anticancer drug-induced increase in P2X levels following inflammation. Oxaliplatin blocks the production of Kir4.1, disrupting the extracellular concentration of K^+^ and leading to an increased likelihood of an AP; (**i**) Flavonoids on Anticancer Drug Effects in Satellite Glial Cells. No relevant research was found on how flavonoids counteract anticancer drug effects on SGCs.

Anticancer drugs exert various effects on the dorsal root ganglion that eventually lead to receptor sensitization, altered channel expression, inflammation, increased likelihood of an action potential and NT release, and oxidative damage (Figure 4a). Inflammation is mediated by increased proinflammatory cytokine production, mainly through the activation of the transcription factor NF-κB (Figure 4b). Flavonoids interfere with different pathways to decrease chemotherapy-induced inflammation (Figure 4c). Icariin, 7,2′,3′/7,2′,4′/–, 7, 3′, 4߰/7,5,4′–trimethoxyflavones and 3′,4′/6,3′/7,2′/7,3′-dimethoxy- flavanol reduce paclitaxel-induced inflammation and allodynia [33,34,35], whereas 6-methoxyflavone mitigates cisplatin-induced static and dynamic allodynia [38]. Moreover, quercetin and rutin restore the mechanical and cold nociceptive thresholds decreased by oxaliplatin [45].

### 4.2. Effects of Anticancer Drugs

#### 4.2.1. Upregulation of the NF-κB Pathway

Paclitaxel activates NF-κB by stimulating TLR4 and upregulating ERK/JNK signaling [46]. These events lead to the phosphorylation and nuclear translocation of NF-κB, increasing the acetylation of the histone H4 and resulting in the transcription of various proinflammatory factors such as CX3CL1, TNF-α, IL-1β, and IL-6 [46] (Figure 4d). Oxaliplatin stimulates NF-κB through the JAK/STAT pathway and causes the production of proinflammatory factors such as CXCL12. Furthermore, this leads to increased MC-P1 (CCL2) transcription in the DRG and its receptor (CCL2R) on macrophages. Activation of CCL2R induces proinflammatory cytokine release, increasing the innate immune response [47]. Cisplatin also uses NF-κB to mediate its proinflammatory effects through the production of nitric oxide and prostaglandins. These factors contribute to inflammation, which leads to membrane depolarization and an increased chance of an AP firing [48].

#### 4.2.2. Increase in Intracellular Ca^2+^

Cisplatin increases N-type voltage-gated Ca^2+^ channel (VGCC) density in small DRG neurons [49] by activating CaMKII [50]. Paclitaxel and oxaliplatin upregulate the α_2_δ_1_ subunit [51,52] (Table 3), which increases VGCC currents, prolongs the neuronal response to mechanical and thermal stimuli, and increases pain [50]. Elevated VGCC mRNA and protein expression [50] increase VGCC density at the presynaptic membrane; therefore, more Ca^2+^ enters the presynaptic terminal. Prostaglandins and bradykinin act on G, upregulating IP3 and increasing intracellular Ca^2+^ release [53]. ATP acts on P2X purinoceptor 3 (P2X3), leading to an increase in intracellular Ca^2+^ [53]. Ca^2+^ activates CaMKII and PKC epsilon, which phosphorylate and activate TRPV1 [53]. Ca^2+^ also increases the fusion of vesicles containing substance P, cGRP, and glutamate with the presynaptic membrane [53] (Figure 4e). The result is an increased probability of postsynaptic action potential generation and a consequent increase in neuropathic pain severity.

#### 4.2.3. Increased GABA Release

The release of more NT by the mechanisms described above increases interneuron activation and GABA release. GABA acts on GABAA receptors on DRG neurons, causing an efflux of Cl^-^ that contributes to the dorsal root reflex, by which the axonal membrane is depolarized in the reverse direction [54]. The reverse ion flow enhances the depolarization of the nociceptor membrane, causing increased release of substance P and cGRP and the consequent release of proinflammatory mediators in a positive feedback loop (see Section 3). In a cisplatin-induced neuropathic pain model, the flavonoid 6-methoxy- flavone increased Cl^-^ influx through GABAA channels [18] and thus decreased presynaptic membrane depolarization and the dorsal root reflex. Smaller amounts of NT were released, decreasing the frequency of action potentials in the postsynaptic neuron and alleviating static and dynamic allodynia [18].

#### 4.2.4. Enhanced Activation of Satellite Glial Cells

Oxaliplatin and taxol contribute to CIPN by activating and increasing satellite glial cells’ coupling (SGC). SGC coupling and increases in GFAP due to ROS production are hypothesized to activate SGCs. Oxaliplatin induces the release of the pro-inflammatory cytokines IL-6 and TNF-α, promoting neuronal hyperexcitability and upregulating the purinergic receptor P2X, increasing the sensitivity to ATP, whose concentration is elevated due to increased action potential firing. Moreover, P2X upregulation increases intra-SGC Ca^2+^ flow and causes intracellular Ca^2+^ waves (ICW) that travel through gap junctions between SGCs and neurons. ICW transmission between SGCs surrounding different neurons causes both SGC and neuronal hyperexcitability [55].

Increased SGC coupling is explained by the oxaliplatin-induced upregulation of Cx-43, a crucial connexon between SGCs. Oxaliplatin also downregulates the inward rectifier channel K_ir_ 4.1, reducing the resting membrane potential and disrupting extracellular K^+^ concentrations. Both of these effects cause the depolarization of SGC-surrounded neurons and thus increase the likelihood of action potential firing (Figure 4h).

#### 4.2.5. Increased Oxidative Stress

Oxaliplatin increases the production of nitric oxide and superoxide, which react to form peroxynitrite. Peroxynitrite is highly reactive and gives rise to nitrogen dioxide and hydroxyl radicals that interact directly with lipids and tyrosine, leading to lipid peroxidation and protein nitrosylation in the DRG ultimately neuronal damage and death through ROS elevation and GSH depletion. Cisplatin accumulation also causes lipid peroxidation and increases inducible nitric oxide synthase (iNOS) activity, and thereby induces oxidative stress and neurotoxicity (Figure 4f). Similarly, paclitaxel induces oxidative stress by increasing DPPH and nitric oxide levels in the DRG [48]. In contrast, thalidomide may cause axonal sensory neuropathy through the depletion of nerve growth factor (NGF). NGF regulates neuronal growth, maintenance, and survival; its depletion is associated with peripheral neuropathy [45].

### 4.3. Flavonoids Counteract the Effects of Anticancer Drugs

#### 4.3.1. Icariin

Icariin activates the sirtuin 1 (S1RT1) pathway, which counteracts paclitaxel-induced NF-κB acetylation of H4 [33]. S1RT1 is a histone deacetylase that may be activated in a NAD-dependent or independent manner. It deacetylates LKB-1, which then activates AMPK. AMPK activates nicotinamide phosphoribosyl- transferase and thus increases NAD+, which is needed for S1RT1 activation [39]. Icariin also directly inhibits NF-κB phosphorylation and nuclear translocation. In contrast, 6-methoxyflavone decreases cisplatin-induced inflammation by inhibiting the enzyme cyclooxygenase 2 (COX-2) [15].

#### 4.3.2. Quercetin, Rutin, and Trimethoxy and Dimethoxy Flavones

Flavonoids alleviate anticancer drug effects by decreasing oxidative stress in the DRG. Quercetin and rutin counteract oxaliplatin’s effects by directly increasing GSH levels and scavenging ROS [56]. Quercetin also decreases the levels of catalase and superoxide dismutase (SOD) and consequently decreases lipid peroxidation and protein oxidation. 7,2′,3′/7,2′,4′/–,7,3′,4′/7,5,4′–trimethoxyflavones and 3′,4′/6,3′/7,2′/7,3′- dimethoxyflavanol also counteract paclitaxel-induced neurotoxicity by scavenging DPPH and nitric oxide [43] (Figure 4g) (Table 4). These flavonoids also inhibit the production of TNF–α and IL-1β [34].

No research on the potential effects of flavonoids on SGCs was found (Figure 4i); thus, future studies should investigate this.

## 5. Flavonoids Counter the Effects of Anticancer Drugs at the Spinal Cord Dorsal Horn

### 5.1. General Effects of Anticancer Drugs and Flavonoids

**Figure 5:**
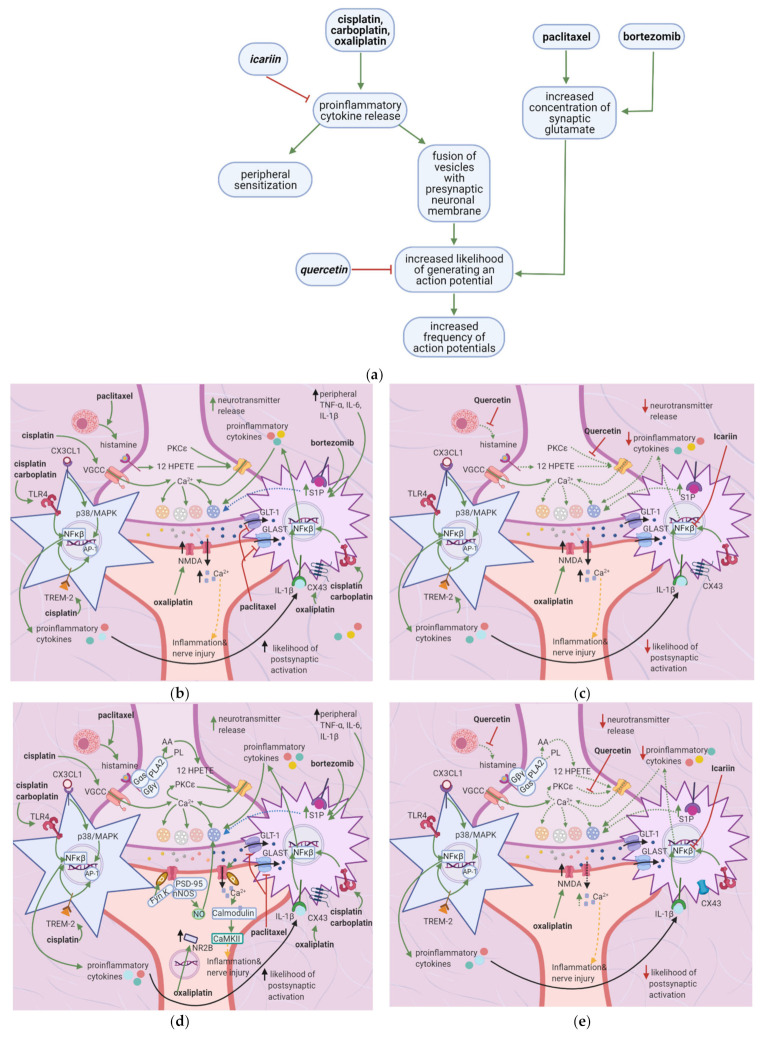
Anticancer drugs modulate signal transmission at a synapse in the dorsal horn spinal cord, and flavonoids counter these effects; (**a**) Simplified Overview of how flavonoids counter the effects of anticancer drugs at the spinal cord dorsal horn. (**b**) Actions of anticancer drugs at the spinal cord dorsal horn. Anticancer drugs increase presynaptic NT release and the likelihood of postsynaptic neuronal activation and activate microglial cells and astrocytes, releasing proinflammatory cytokines. Proinflammatory cytokines increase glutamate release and sensitize primary afferent channels; (**c**) Flavonoids counteract anticancer drugs’ actions at the dorsal horn by inhibiting mast cell degranulation, PKC epsilon translocation, and NF-κB activation. This decreases the likelihood of postsynaptic action potential generation; (**d**) Anticancer drugs increase intracellular Ca^2+^ at the synaptic terminal in DRG neurons via activation of VGCC and histamine release, which leads to TRPV1 sensitization; (**e**) Flavonoids reduce neuropathic pain by inhibiting mast cell degranulation. Thus, histamine release and H1R activation decrease, eventually leading to decreased NT release.

Cisplatin and carboplatin increase proinflammatory cytokine release by astrocytes and microglial cells [7,64], contributing to peripheral sensitization. Oxaliplatin similarly enhances cytokine release and peripheral sensitization by increasing astrocyte coupling [65]. Paclitaxel and bortezomib increase synaptic glutamate concentrations [66,67], increasing the likelihood of action potential generation in the postsynaptic neuron (Figure 5a). Increased postsynaptic action potential frequency intensifies neuropathic pain. Quercetin reverses the paclitaxel-induced decrease in thermal hyperalgesia and mechanical allodynia thresholds [41], while icariin alleviates paclitaxel-induced mechanical allodynia and spinal neuroinflammation [42].

### 5.2. Effects of Anticancer Drugs

Cisplatin and carboplatin are ligands for TLR4 [7,64], and cisplatin upregulates the TREM-2 ligand [68] (Table 5). Subsequent activation of the TLR4 and TREM-2 pathways eventually results in NF-κB activation, culminating in proinflammatory cytokine release by astrocytes and microglial cells [69]. Chemokines released by DRG neurons, such as CX3CL1, also cause NF-κB activation and proinflammatory cytokine release [7,70] (Figure 5b). Proinflammatory cytokines cause peripheral sensitization via the processes mentioned in Section 2; IL-1β acts on IL-1R on astrocytes, stimulating NF-κB activation and hence the release of more proinflammatory cytokines [71]. These cytokines also stimulate the fusion of presynaptic glutamate vesicles with the DRG neuron membrane [67].

Paclitaxel inhibits GLAST and GLT-1 (glutamate uptake channels) located on astrocyte membranes [66]. Bortezomib increases sphingolipid metabolism, resulting in increased synthesis of S1P receptors [67]. Alternatively, S1P is upregulated by increases in peripheral TNF-α, IL-1β, and IL-6, commonly seen in neuropathic pain states. Increased S1P is correlated with increased presynaptic membrane fusion of glutamate vesicles [67]. These changes increase synaptic glutamate levels, upregulating NMDA and AMPA on the postsynaptic membrane. They contribute to neuropathic pain by increasing the postsynaptic neuron’s depolarization and consequently increasing the chance of generating an action potential.

Oxaliplatin upregulates the CX43 gap junctional protein, increasing coupling between astrocytes [65]. This increases astrocyte activation and proinflammatory cytokine release. Furthermore, histamine released by mast cells acts on HIR; the subsequent cascade results in arachidonic acid production. Arachidonic acid activates 12 HPETE, which in turn sensitizes TRPV1 (Figure 5d) [41]. More Ca^2+^ enters the presynaptic neuron, causing more vesicle fusion and NT release, thereby increasing the likelihood of generating a postsynaptic action potential, increasing neuropathic pain intensity.

### 5.3. Flavonoids Counter the Effects of Anticancer Drugs

#### Quercetin and Icariin

The flavonoid quercetin inhibits mast cell degranulation and PKC epsilon movement to the membrane, thus decreasing the activation of TRPV1 (Figure 5e) [41]. This reduces Ca^2+^ entry and thus the fusion of vesicles with the membrane, decreasing the likelihood of a postsynaptic action potential. Icariin inhibits NF-κB in the spinal cord dorsal horn (Figure 5e) (Table 6); thus, fewer proinflammatory cytokines are released, reducing the fusion of vesicles with the membrane. Overall, the frequency of action potentials in the postsynaptic neuron decreases, decreasing neuropathy intensity [42] (Figure 5c).

## 6. Flavonoids Counter the Effects of Anticancer Drugs on Astrocytes and Microglial Cells at the Spinal Cord Dorsal Horn

### 6.1. General Effects of Anticancer Drugs and Flavonoids

**Figure 6:**
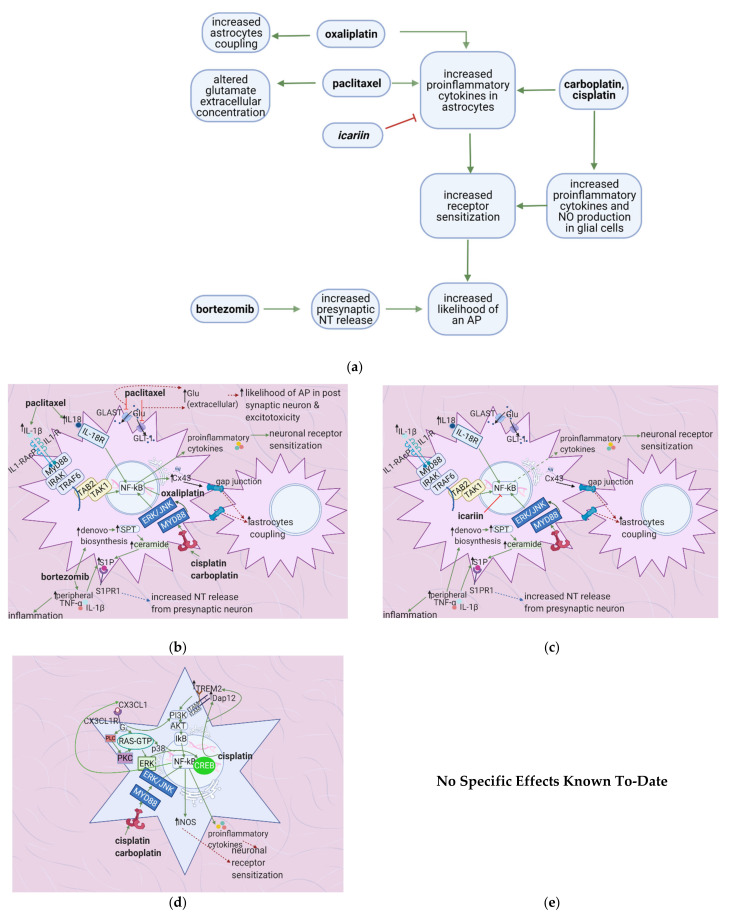
The actions of anticancer drugs (left) and flavonoids (right) on astrocytes and glial cells. (**a**) Simplified Overview of how flavonoids counteract the mechanisms of anticancer drugs at the level of astrocytes and microglial cells (**b**) Anticancer Drugs on Astrocytes. Anticancer drugs activate NF-κB and consequently lead to the production of proinflammatory cytokines that sensitize neuronal receptors. Bortezomib increases the levels of S1P, which is hypothesized to increase presynaptic NT release. Paclitaxel blocks glutamate receptors, thus increasing its extracellular concentration and increasing the likelihood of an AP in the post-synaptic neuron. (**c**) Flavonoids on Anticancer Drug-Induced Increases in Proinflammatory Cytokines in Astrocytes. Icariin inhibits NF-κB and thus counteracts the increase in proinflammatory cytokine levels, decreasing neuronal sensitization and inflammation. (**d**) Anticancer Drugs on Glial Cells. Anticancer drugs activate NF-κB and CREB, leading to proinflammatory cytokines and NO production, ultimately causing neuronal receptor sensitization. (**e**) No specific effects of flavonoids on microglial cells are elucidated to-date. Studies usually investigate the spinal cord dorsal horn as a whole.

Bortezomib causes astrocyte activation and glutamate release by upregulating the S1P pathway; this leads to peripheral sensitization and an increased likelihood of postsynaptic action potential generation [67]. Cisplatin upregulates TREM2/DAP12 and TLR4 signaling [57,64,69], causing proinflammatory cytokine release and thus peripheral sensitization. Vincristine exerts the same effects by upregulating Iba-1, CX3CR1, and p–p 38 [72]. Oxaliplatin and paclitaxel enhance astrocyte activation, increasing the likelihood of postsynaptic action potential generation [63] (Figure 6a). Astragli radix decreases astrocyte and microglial activation and oxaliplatin-induced neuronal damage, while icariin reduces proinflammatory cytokine release and astrocyte activation [42,73].

### 6.2. Effects of Anticancer Drugs

#### 6.2.1. Upregulation of the S1P Pathway

Bioactive sphingolipid metabolites are potent signaling molecules involved in bortezomib-induced CIPN. Bortezomib affects the S1P signaling pathway by increasing ceramide and its biosynthetic precursors such as S1P (Figure 6b). As astrocytes express S1PR1 (at higher levels than glial cells), they mediate bortezomib-induced CIPN; S1P triggers them to become reactive (Table 7). This form of astrocyte activation is associated with increases in GFAP, TNF, and IL-1β. S1PR1-induced inflammation establishes a feed-forward mechanism that dysregulates sphingolipid production, as TNF and IL-1β cause the activation of enzymes in the ceramide and S1P pathways. S1PR1 also increases glutamate release, which sustains neuropathic pain [67].

#### 6.2.2. Upregulation of the TLR4 Pathway

Cisplatin activates TLR4 receptors via the MYD88/ NF-κB pathway, leading to the release of inflammatory cytokines like TNF and consequent mechanical allodynia. Activating transcription factor 3 (ATF3), whose concentration increases during inflammation, augments TLR4 signaling to NF-κB. This pathway is also seen in microglia [64].

Similarly, paclitaxel exerts its effects through TLR4 signaling–a pathway shared by IL-1 receptors, which are activated by proinflammatory cytokines elevated by paclitaxel as shown (see Figure 6b). Both TLR4 and IL-1 receptors can activate MyD88 dependent cascades, leading to early NF-κB activation. MyD88 is an adaptor protein that links to the IL-1 receptor-associated kinase (IRAK); TNF receptor-associated factor 6 (TRAF6) lies downstream. When TRAF6 is activated, it becomes ubiquitinated and interacts with TAB1, TAB2, and the kinase TAK1, activating another kinase, IKK-2. IKK-2 then phosphorylates I-κB, causing NF-κB phosphorylation and nuclear translocation [42,57].

#### 6.2.3. Increased Expression of Cx-43

Besides upregulating GFAP (as with paclitaxel), oxaliplatin upregulates Cx-43, a component of the gap junctions between astrocytes. Cx-43 mediates the exchange of ions, metabolites, glia-transmitters, and the propagation of Ca^2+^ waves between astrocytes. It increases astrocyte coupling and activation and consequently plays a role in oxaliplatin-induced hypersensitivity [63].

#### 6.2.4. Downregulation of Glutamate Uptake Receptors and Increased GFAP

Paclitaxel activates astrocytes and not microglial cells. As glutamate is a major excitatory NT, regulation of its uptake is essential. Paclitaxel downregulates the glial glutamate receptors GLAST and GLT-1 on astrocytes. Consequently, paclitaxel induces nociceptive behaviors and hypersensitivity to peripheral thermal and mechanical stimuli due to glutamate’s impaired clearance in the inter-synaptic space [56]. Paclitaxel also increases the expression of GFAP, further supporting the astrocyte activation hypothesis in parallel with hypersensitivity [42].

#### 6.2.5. Enhanced TREM2/DAP12 Signalling

Cisplatin enhances the triggering receptor’s expression on myeloid cells 2 (TREM2) /DNAX-activating protein of 12 kDa (DAP12) signaling in spinal microglia. TREM2, from the immunoglobulin/lectin superfamily, associates with DAP12 to mediate intracellular interaction through the phosphorylation of tyrosine residues in its immunoreceptor tyrosine activation motif (ITAM) motifs. ITAM later activates phosphatidylinositol 3 kinase (PI3K) and downstream effectors until NF-κB is active, increasing proinflammatory cytokines such as IL-1β, IL-6, and TNF-α, iNOS, and CD16 [69].

#### 6.2.6. Increased CX3CL1 Expression

CX3CL1 is a chemokine that stimulates spinal microglia by acting in its G-protein coupled receptor CX3CL1R to release pain mediators. Vincristine upregulates Iba-1, CX3CR1, and p-p38, which eventually activate NF-κB and CREB. NF-κB and CREB activate the microglia, which release proinflammatory cytokines (TNF-α and IL-1β) (Figure 6d). Soluble CX3CL1 activates the microglia-specific receptor CX3CR1, causing the phosphorylation of p38 MAPK, thereby promoting proinflammatory cytokines’ secretion. Targeting of the Notch signaling pathway inhibits this CX3CL1/p38 signaling pathway through a mechanism not entirely elucidated [72]. No specific effects of flavonoids on spinal microglial cells are known to date (Figure 6e); thus, this is an area of investigation of future research.

### 6.3. The Flavonoids Icariin and Astragli Radix Counter the Effects of Anticancer Drugs

Icariin counteracts paclitaxel-induced GFAP expression and thus represses astrocyte activation. Icariin also inhibits the production of proinflammatory cytokines such as TNF-α, IL-1β, and IL-6 in the spinal cord [42] (Table 8) (Figure 6c). In addition to isolated flavonoids, Astragali radix is an adaptogenic herbal product that improves chemotherapy patients’ quality of life. Astragai radix is rich in various phytochemicals, including isoflavonoids, while isoflavones are more concentrated in hydroalcoholic extracts of Astragali radix when compared with aqueous. Besides, 50% hydroalcoholic extracts of Astragali radix reduced ATF-3 nuclear immunoreactivity in L4-L5 DRG, oxaliplatin-induced molecular and morphometric alterations in peripheral nerve and dorsal root ganglia, and the activation of microglia and astrocytes in a Sprague-Dawley rat model of oxaliplatin-induced neurotoxicity [73].

## 7. Flavonoids Counter Neuronal Injury Induced by Anticancer Drugs

### 7.1. General Effects of Anticancer Drugs and Flavonoids

**Figure 7:**
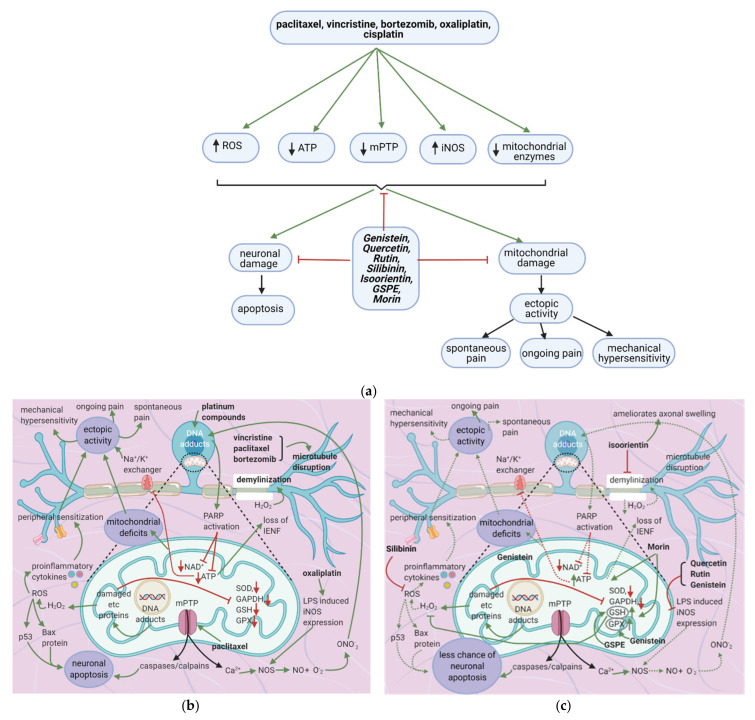
Anticancer drugs cause neuronal injury, and flavonoids counter these effects; (**a**) Simplified Overview of how flavonoids counter neuronal injury induced by anticancer drugs (**b**) Mechanisms of neuronal injury by anticancer drugs. Mitochondrial damage and sensitization of primary afferent channels cause ectopic discharges, which contribute to spontaneous pain, ongoing pain, and mechanical hypersensitivity; (**c**) Flavonoids alleviate neuronal damage caused by anticancer drugs, reducing spontaneous pain, mechanical hypersensitivity, and the likelihood of apoptosis.

Anticancer drugs cause mitochondrial and neuronal damage [76,77] and upregulate iNOS [56]. Flavonoids restore ATP levels and mitochondrial protective enzymes, reverse neuronal damage, and decrease ROS production [78,79,80,81] (Figure 7a). Thus, they reduce spontaneous pain, ongoing pain, and mechanical hypersensitivity.

### 7.2. Effects of Anticancer Drugs

#### 7.2.1. Mitochondrial Damage

Paclitaxel increases the activity of the mitochondrial permeability transition pore (mPTP) channel [76] (Table 9). Consequently, more Ca^2+^, caspases, and calpains leave the mitochondria; caspases and calpains trigger neuronal apoptosis [77]. Furthermore, paclitaxel, vincristine, and bortezomib disrupt microtubules (Figure 7b) [82,83]. Paclitaxel causes microtubules to polymerize and inhibits their depolymerization [84]. Platinum compounds cause DNA adducts to form within mitochondria and the neuronal nuclei [85]. Nuclear DNA adducts activate PARP, decreasing mitochondrial NAD^+^ and ATP and causing further mitochondrial damage [86]. Under these low ATP conditions, intraepidermal nerve fibers are lost [87].

#### 7.2.2. Neuronal Damage

Furthermore, the activity of the Na^+^/K^+^ exchanger will decrease, leading to increased ectopic activity [88]. Ectopic activity leads to spontaneous pain, ongoing pain, and mechanical hypersensitivity [76]. Mitochondrial DNA adducts damage the electron transport chain’s proteins, increasing oxidative stress and H_2_O_2_ release [76]. H_2_O_2_ causes ROS formation and demyelinates the neuron; ROS causes p. 53 and Bax release, leading to neuronal apoptosis [77]. ROS also increases the release of proinflammatory cytokines, which cause peripheral sensitization, further contributing to ectopic activity [89]. Damage to electron transport chain proteins causes the downregulation of protective enzymes such as SOD, GAPDH, GSH, and GPX, leading to excessive ROS production [76].

#### 7.2.3. Enhanced iNOS Expression

Oxaliplatin increases LPS-induced iNOS expression [56]. Ca^2+^, together with iNOS, leads to the production of NOS, which increases NO. NO combines with oxygen free radicals to form ONO^-^_2,_ enhancing DNA adducts production [56].

### 7.3. Flavonoids Counter the Effects of Anticancer Drugs

The flavonoids morin and GSPE restore GSH levels, and morin restores ATP levels (Figure 7c) [78,79]. Increased ATP levels restore the activity of the Na^+^/K^+^ exchanger, decreasing ectopic activity and thus neuropathic pain, while elevated GSH levels reduce H_2_O_2_ and ROS production. These effects reduce the chance of apoptosis and also decrease ectopic activity. Thus, spontaneous pain, ongoing pain, and mechanical hypersensitivity will all decrease. Genistein restores mitochondrial GPX levels while isoorientin ameliorates axonal swelling and prevents demyelination [80,81]. Quercetin, rutin, and genistein reduce LPS-induced iNOS expression [56,81] and thereby reduce ONO_2_^-^ production and DNA adduct formation. Besides, the natural flavonoid silibinin prevents oxidative damage and exerts antineuropathic effects in a rat model of painful oxaliplatin-induced neuropathy [90] (Table 10).

## 8. Flavonoids: Promise, Applications, and Side Effects

CIPN is a multifactorial disease with various pharmacological mechanisms. Effective treatments should influence the mechanisms that contribute most to the symptoms. Damage to DRG neuronal cell bodies or axons contributes most strongly to CIPN symptom development. Likewise, flavonoids with the most success in animal models affect these areas by reducing peripheral sensitization of DRG neurons, modulating synaptic transmission at the spinal dorsal horn, and reducing mitochondrial damage in DRG neurons, among other mechanisms. However, how these mechanisms interact with and influence each other to cause symptoms still requires extensive investigation.

Moreover, pain results from interactions between central and peripheral mechanisms [93,94]. More information about central-peripheral interaction and central nervous system mechanisms of pain transmission (neuromodulators, neuroplasticity, central sensitization, and NTs) is needed to understand the pathophysiology of CIPN [50] entirely. Also, there are many clinical phenotypes of CIPN, and each requires a specific standardized treatment approach. For example, oxaliplatin and paclitaxel induce neuropathy through different mechanisms [93]. Adding a different complexity level, combinations of different anticancer drugs are used in treatment regimens [4,95].

Moreover, a specific flavonoid will alleviate some, but not all, symptoms. For example, 6-methoxyflavone [18] alleviates static and dynamic allodynia, whereas dimethoxyflavonol inhibits tactile allodynia [44], and quercetin decreases the thermal hyperalgesia and mechanical allodynia thresholds [41]. Therefore, since CIPN involves many symptoms, and specific flavonoids counteract specific anticancer drugs, combination therapies warrant further investigation [3].

It is essential to consider the concentrations of flavonoids that are useful in the treatment of CIPN. Flavonoids are present in relatively low concentrations in fruits and vegetables; these sources also contain a mixture of secondary plant metabolites such as vitamin C, folate, potassium, and fiber [96]. These secondary metabolites have known health benefits that cannot be replaced by a single compound (e.g., flavonoids) given as a dietary supplement [96]. Suppliers of flavonoid supplements recommend daily doses many times higher than those found in a flavonoid-rich diet. For example, quercetin is offered as a supplement with daily doses of 1 g or more [96], while its daily dietary intake is estimated to be between 10–100 mg [96].

Other issues to be considered in evaluating flavonoids as dietary supplements include drug interactions, trace element chelation, and thyroid status [97]. In vitro experiments indicate that purified flavonoids and flavonoid-rich extracts chelate iron, posing a risk for iron deficiency individuals. Flavonoids also interact with copper, manganese, and vitamin C [97,98]. They may exhibit antithyroid and goitrogenic activity. For example, quercetin and isoflavones inhibit iodothyronine deiodinase activity. High-dose isoflavones inhibit thyroid hormone biosynthesis, have estrogenic effects, and are goitrogenic [99,100]. Flavonoids may interfere with the absorption, tissue distribution, metabolism, and excretion of classical xenobiotics due to similar metabolic pathways. Notably, flavonoids interfere with all phase II enzymes, affecting the organism’s ability to detoxify endogenous and exogenous xenobiotics. For example, quercetin and kaempferol increase either the transcription or activity of the enzyme UDP-glucosyltransferase A1 [101].

Another vital factor to consider while reviewing in vitro studies is the effect of flavonoid distribution on local concentrations and drug interactions. For instance, quercetin and kaempferol inhibit CYP3A4 and, consequently, the metabolism of the Ca^2+^ channel blockers nifedipine and felodipine in human liver microsomes at concentrations >10 μmol/L [102]. On the other hand, quercetin did not inhibit CYP3A4 metabolism of the statin simvastatin in pigs. A possible explanation for this is a lower hepatic concentration than observed in vitro [103]. Flavonoids (with C5 hydroxy and methoxy groups [104,105]) inhibit ABC transposers. The inhibition has positive consequences for poorly absorbed drugs but may result in drug toxicities for low therapeutic index drugs [106].

Given these side effects, it has been concluded by some that whole fruits and vegetables are more beneficial to health than any single plant constituent [107]. On the flip side, overexpression of ABC transporters is one of the major mechanisms of multidrug resistance encountered during chemotherapy treatments. Cancer cells overexpress the ABC transporter, which pumps out anticancer drugs before they can have a significant effect. Thus, flavonoids can reduce drug resistance and thereby enhance the efficacy of chemotherapy. Moreover, several studies suggest that flavonoids sensitize cancer cells to chemotherapy [108,109]. Quercetin especially has promise, combined with vincristine, to increase breast cancer treatment efficacy [110]. Therefore, the potential of flavonoids as adjuncts in chemotherapy creates an additional incentive to investigate further their potential in counteracting CIPN, especially in studies involving humans.

Flavonoids have potential as therapeutic agents for preventing CIPN; however, many questions remain unanswered due to the lack of flavonoid studies with human subjects; e.g., what serum concentration must be achieved to get a significant therapeutic effect? Can this concentration be achieved by supplementing the diet with flavonoid-rich foods, or are intravenous injections a must? According to Mongiovi et al., increasing citrus fruit intake poses its own problems in patients undergoing chemotherapy—their results showed a positive association between citrus fruit intake (rich in flavonoids) and neuropathic pain symptoms [111], which may indicate potential detrimental interactions between flavonoids and other substances in citrus fruits. However, other studies evaluating the effects of diet on CIPN did not show this association [112], and thus more studies are required to further elucidate the effects of citrus fruits on CIPN symptoms. Furthermore, due to flavonoids’ potential side effects, an extensive cost-benefit analysis in humans is needed to determine whether a flavonoid-based treatment should be explored further.

Translating results obtained from mouse models to humans presents challenges of its own. Sex, differences in social structure, and variations in genotype and neuroanatomy all influence pain pathways and pain perception [3,113]. Due to the differences in symptoms experienced by humans and mice [113], the effects of flavonoids on humans are expected to be highly variable. Another complicating factor is that, unlike patients with CIPN, most mice in these studies do not have cancer. Furthermore, murine chemotherapy delivery methods may not match clinical ones; additionally, the sex of the animals does not match clinical demographics–studies with animals use mostly male mice, but there are many female cancer patients as well [3]. Also, in animal models, only acute (experienced within the first six months after treatment), not chronic (occurring about two years following treatment), neuropathic pain is studied [3]. Thus, the elucidated mechanisms are related to the acute phase only, and treatment with flavonoids may not have the same effect on chronic neuropathic pain [3]. The mechanisms of chronic neuropathic pain warrant more research in animal models, especially in the context of flavonoids. The symptoms of chronic neuropathic pain vary from those of the acute phase. While the acute phase is marked by dysesthesia, paraesthesia, and hyperesthesia, chronic neuropathy is mainly associated with sensory ataxia, insomnia, anxiety, depression, cognitive and functional deficits, and fall risk [10,11,114]. So far, only one study has assessed the effects of flavonoids on symptoms specific to chronic neuropathy: Chtourou et al. investigated cisplatin exposure to acetylcholinesterase, ATPase, and oxidative stress biomarkers and the potential association this may have on behavioral performance in aged rats. The protective mechanisms of the flavonoid naringin were also studied. While cisplatin decreased enzymatic and non-enzymatic antioxidant activity in the hippocampus and raised levels of ROS, NO, MDA, and PCO, naringin reversed these effects and alleviated cisplatin-induced cognitive deficits (as seen by improved performance on the behavioral test administered) [115]. Flavonoids thus reverse anticancer drug-induced cognitive decline in chronic CIPN; in this light, their effects on the central nervous system merit further investigation. Also, flavonoids may hold promise in treating depression linked to chronic CIPN, since they are effective as antidepressants due to their antioxidant activity [116]. Furthermore, recent clinical results indicate that pediatric patients receiving azole antifungal treatment along with one-hour infusions of vincristine develop less severe peripheral neuropathy than patients receiving only vincristine. Similar studies should be conducted with flavonoids, in which isolated or mixed flavonoids are delivered during chemotherapy treatment to decrease the onset and severity of symptoms [117].

In short, increasing evidence indicates that flavonoids may alleviate the symptoms of both acute and chronic CIPN, increase the efficacy of chemotherapy, and reduce the cognitive dysfunction that results from it; thus, their side effects, effects in humans, and mechanisms of action are priorities for further investigation. 

## 9. Conclusions

Flavonoids hold great promise in the management of CIPN. Investigating the mechanisms through which flavonoids act furthers the understanding of peripheral neuropathy and offers new methods to overcome it. The burden of CIPN and the promise of flavonoids encourages future research into their actions in humans, as well as their therapeutic index and side effects.

## Figures and Tables

**Table 1:** Mechanisms of CIPN at the peripheral nociceptor induced by anticancer therapy.

Mechanism of CIPNP	Neuropathic Pain Model	Mode of Administration/Concentration	Animal Model	Reference
Increased macrophage infiltration	Paclitaxel and bortezomib induced	(in vitro) IV bortezomib 0.2 mg/kg, 3 times a week for 8 weeks (in vitro) 2 doses of 18 mg/kg paclitaxel given 3 days apart	Female Wistar rats Adult Male Sprague Dawley Rats	[30,31]
Sensitization of TRPA1 via increased production of ROS, RNS, and RCS.	Oxaliplatin induced Cisplatin induced Paclitaxel induced	Intraperitoneal/3 mg/kg Intravenous/ 2 mg/kg Intraperitoneal/3 times per week for 5 weeks (2 mg/kg) Intraperitoneal/6 mg/kg	Male Dunkin-Hartley guinea pigs, male Sprague-Dawley rats, male C57BL/6 mice, wild-type (Trpa1+/+) or TRPA1-deficient mice (Trpa1–/–) Male C57BL/6 mice, wild-type (Trpa1 +/+), or TRPA1-deficient mice (Trpa1 –/–)	[32,33]
Increased expression of FKN, which binds to CX3CR1, increasing ROS production and enhancing trafficking of macrophages to the sciatic nerve. ROS activated TRPA1, evoking pain.	Vincristine induced	Intraperitoneal/0.5 mg/kg for two 5 day cycles	Adult male and female C57BL/6 J mice	[34]
Increased production of tryptase, which cleaves PAR2, causing increases in PKC∈ and PKA. PKA sensitizes TRPV1, TRPV4 and TRPA1, whereas PKC ∈ sensitizes TRPV1 and TRPV4.	Paclitaxel induced	Intraperitoneal/ Four doses of 1 mg/kg every two days	Male ICR mice	[35]
IL-1β increases TTXR Na^+^ currents via the p38/MAPK pathway.	Isolated DRG cells	IL-1β (10 ng/mL) applied using multibarrel fast drug delivery system	Male Sprague-Dawley Rats	[36]
TNF- α increases TTXR Na^+^ currents via the p38/MAPK pathway	Isolated DRG cells	(in vitro) recombinant murine TNF-α (50 µg/mL) solution (in vivo) 1ng of TNFα- in 10 µL injected into rat hind paw plantar surface	ICR adult male mice	[37]
Application of IL-1β, TNF-α, and IL-6 on peripheral nociceptors dose-dependently led to cGRP release	Incubated skin flaps	(in vitro) murine TNF-α (0.05–500 ng), murine IL-1β (0.02–200 ng), human IL-8 (0.1 ng to 1 μg), mIL-6 (0.02–200 ng)	Male Wistar rats	[38]
Increased expression of TRPA1 and TRPV1 in small sized DRG neurons and TRPM8 in medium sized DRG neurons.	Oxaliplatin induced	(in vitro) Intraperitoneal One dose of 6 mg/kg	Male Wistar rats	[39]
Increased expression of HCN and decreased expression of TREK1 and TRAAK channels.	Oxaliplatin induced	(in vitro) Intraperitoneal 3 injections (1,3,6 mg/kg)	Male C57BL6J mice	[40]

**Table 2:** Role of flavonoids in countering CIPN mechanisms at the peripheral nociceptor.

Flavonoid	Neuropathic Pain Model	Animal Model	Flavonoid Concentration	Mechanism-based Intervention	Effect on Neuropathy	Reference
Quercetin	Paclitaxel induced	Adult male Sprague-Dawley rats and Institute of Cancer Research mice	3, 10 and 30 μmol/L (in vitro) Intragastric administration of 20 mg/kg or 60 mg/kg once per day for 40 days for rats and 12 days for mice (in vivo)	Inhibited degranulation of mast cells, PKC epsilon translocation from the cytoplasm to the cell membrane	Dose-dependent increase of thermal hyperalgesia and mechanical allodynia thresholds	[41]
Icariin	Paclitaxel induced	3 to 4 month old male Sprague Dawley Rats	(in vitro and in vivo) 25, 50,100 mg/kg	Reduction of IL-1 β, TNF-α and IL-6 release from the DRG, astrocytes, and microglia	Decreased mechanical allodynia and spinal neuroinflammation	[42]
Trimethoxy flavones	Paclitaxel induced	Adult swiss Albino mice of either sex	(in vitro and in vivo) 25, 50, 100 or 200 mg/kg	Concentration-dependent decrease of IL-1β, TNF-α, and free radicals	Dose-dependent decrease of tactile allodynia, thermal hyperalgesia, and cold allodynia	[43]
Dimethoxy flavones	Paclitaxel induced	Male Swiss Albino Mice	(in vitro and in vivo) 25, 50, 100 or 200 mg/kg	Concentration-dependent decrease of IL-1β, TNF-α, and free radicals	Dose-dependent decrease of tactile allodynia, thermal hyperalgesia and cold allodynia	[44]

**Table 3:** Previous studies investigating the mechanisms by which anticancer drugs exert their effects on the DRG.

Mechanism of CIPNP	Neuropathic Pain Model	Mode of Administration/ Concentration	Animal	Reference
Increased VGCC current density in DRG neurons via CaMKII; Increased VGCC protein levels	Cisplatin induced	5 mg/kg (in vivo), 0.5 μM and 5 μM (in vitro)	Male and female Wistar rats for in vitro procedures. Male Sprague-Dawley rats for in vivo procedures	[49]
Upregulation of VGCC α_2_δ_1_ subunit in DRG	Paclitaxel induced	IP/4 mg/kg single injection; 4 mg/kg administered 4 times on alternate days Intravenous/ 4 mg/kg single injection	Male ddY mice	[57]
Increased phosphorylation of STAT3; increased levels of CXCL12 mRNA and protein	Oxaliplatin induced	IP/5 injections of 4 mg/kg each, administered on consecutive days	Male Sprague–Dawley rats	[58]
Upregulation of p65 mediated CX3CL1 expression in DRG	Oxaliplatin induced	Intraperitoneal/ 5 injections of 4 mg/kg each, administered on consecutive days	Male Sprague-Dawley rats	[59]
Downregulation of SIRT1 expression and an increase in histone acetylation; induction of NF-κB(p65) activation and nuclear translocation; upregulation of proinflammatory factors (TNF-α, IL-1b, IL-6 ); Activation of astrocytes	Paclitaxel induced	IP/8 mg/kg per day for 3 consecutive days	Male Sprague Dawley rats	[42,43]
increased lipid peroxidation and protein nitrosylation; increased inducible nitric oxide synthase.	Oxaliplatin induced	IV/1 mg/kg dose twice a week (total of nine injections).	Male Swiss mice	[56]
Increased TNF-α, IL-1β, DPPH, and NO.	Paclitaxel induced	IP/A single dose (10 mg/kg)	Male Swiss albino mice	[44]
Stimulates COX-2 expression	Cisplatin induced	IP/3 mg/kg once a week for four consecutive weeks.	Male Sprague-Dawley rats	[48]
TLR4 signaling in the spinal cord dorsal horn and DRG induces and maintains CIPN	Paclitaxel induced	IP/4 injections of 2 mg/kg administered every other day	Male Sprague-Dawley rats	[60]
Upregulation of CX3CL1 via NF-κB–dependent H4 acetylation	Paclitaxel induced	IP/3 injections of 8 mg/kg, on 3 alternate days	Male Sprague-Dawley rats	[46]
Increased expression of CCL2/CCR2 leading to innate immune response	Oxaliplatin induced	IP/1 injection of 3mg/kg	Male Sprague-Dawley rats	[47]
Increased the expression of TRPV1	Oxaliplatin induced	IP/I injection of 6 mg/kg	Male Wistar rats	[39]
Increased VGCC expression mediated by CaMKII	Oxaliplatin induced.	In vitro and in vivo w/ variant conc	Wistar rats	[61]
Increased gap-junctional coupling among SGCs; increased GFAP production	Taxol and Oxaliplatin induced	Oxaliplatin–IP/ 2 injections of 4 mg/kg–3 days apart. Taxol- IP/ 2 injections of 18 mg/kg–3 days apart	Balb/c mice	[62]
increased ROS, GFAP, and Cx-43 decreased Kir4.1 channels	Oxaliplatin induced	in vitro/ 1 and 10 μM for 2,4 and 24 h.		[63]
Peripheral neuropathic pain associated with increase in ∝2δ1 subunit in spinal cord	Paclitaxel induced Oxaliplatin induced	In vitro/Intraperitoneal 2 mg/kg paclitaxel on 4 alternate days In vitro/ 6 mg/kg oxaliplatin intraperitoneal	Adult male Sprague Dawley Rats Male Wistar rats	[51,52]
Increased N-type VGCC density in small DRG neurons	Cisplatin induced	In vitro/0.5 μM and 5 μM incubated for 24 or 48 h.	Male and female Wistar rats	[49]

**Table 4:** Previous studies investigating the effects of flavonoids on CIPN models at the DRG.

Flavonoid	Neuropathic Pain Model	Animal	Mode of Administration/ Concentration of Flavonoid	Mechanism-Based Intervention	Effect on Neuropathy	Reference
Icariin	Paclitaxel induced	Male Sprague Dawley rats	IG/25, 50, 100 mg/kg.	Activated SIRT1 via histone acetylation; Prevented NF-κB(p65) phosphorylation and nuclear translocation; prevented the production of TNF-α, IL-6, and IL-1β; Suppressed astrocyte activation.	Alleviated mechanical allodynia. (100 mg/kg in the long term) and spinal neuroinflammation.	[42]
Rutin and quercetin	Oxaliplatin	Male Swiss mice	IP/ rutin or quercetin (25, 50, and 100 mg/kg) 30 min before every oxaliplatin injection (1 mg/kg).	Decreased Fos expression; Decreased nitrotyrosine and iNos expression, and lipid peroxidation.	Inhibited the decrease in mechanical (a.d) and cold nociceptive threshold. Prevented the shrinkage of dorsal horn neurons	[56]
7, 2′, 3′/7,2′, 4′/–,7,3′,4′/7, 5,4′–trimethoxy flavone	Paclitaxel	Male and female adult Swiss albino mice	SC injection/ 25, 50, 100 and 200 mg/kg.	Inhibition of TNF–α, IL–1β (d.d). Scavenging DPPH; Preventing NO generation (d.d)	Alleviated tactile allodynia, cold allodynia and thermal hyperalgesia in mice	[43]
3′,4′/6,3′/7,2′/7,3′-dimethoxy flavonol	Palcitaxel	Male Swiss albino mice	SC/25, 50, 100, and 200 mg/kg	Decreased TNF-α, IL-1β (d.d); Scavenged DPPH and NO (d.d)	Improved tactile allodynia, cold allodynia and thermal hyperalgesia(d.d)	[44]
6-Methoxyflavone	Cisplatin	Male Sprague-Dawley rats	Intraperitoneal/25, 50 and 75 mg/kg Also conducted in silico and in vitro studies	Inhibits COX-2; Stimulates GABAA channels	Improved static and dynamic allodynia	[48]

**Table 5:** Studies investigating the mechanisms by which anticancer drugs exert their effects.

Mechanism of CIPN	Neuropathic Pain Model	Mode of Administration/Concentration	Animal	Reference
Strong TREM2/DAP12 signaling continuously activated microglial cells, which resulted in neuropathic pain.	Cisplatin induced	Intraperitoneal/Accumulated dose of 23 mg/kg delivered in 2 rounds daily for 5 days with a 5 day break between rounds. (in vitro and in vivo)	Adult male mice, 9–10 weeks old	[68]
Oxaliplatin upregulates spinal CX3CLI, causing central sensitization and acute CIPN	Oxaliplatin induced	Intraperitoneal/single dose of 4 mg/kg (in vitro and in vivo)	Male Sprague Dawley rats	[70]
Increased S1P, S1PR1, and dihydro-S1P due to dysregulated sphingolipid metabolism	Bortezomib induced	(in vitro and in vivo)	Male Sprague Dawley rats, S1pr1 knockout and knockdown mice	[67]
Downregulation of GLAST and GLT-1 on astrocyte membranes	Paclitaxel induced	Intraperitoneal/4 injections of 2 mg/kg every other day (in vitro)	Adult Male Sprague Dawley Rats, 8–10 weeks old	[66]
Upregulation of CX43 gap junctional proteins in astrocytes in the spinal cord dorsal horn	Oxaliplatin induced	Intraperitoneal/4 injections of 2mg/kg each given every other day. (in vitro and in vivo)	Male Sprague Dawley rats	[65]

**Table 6:** Studies investigating the effects of flavonoids on CIPN models.

Flavonoid	Neuropathic Pain Model	Animal	Mode of Administration/ Concentration of Flavonoid	Mechanism-based Intervention	Effect on Neuropathy	Reference
Quercetin	Paclitaxel induced	adult male Sprague-Dawley rats and mice	3, 10 and 30 μmol/L (in vitro) Intragsteral administration of 20 mg/kg or 60 mg/kg once per day for 40 days for rats and 12 days for mice (in vivo)	Inhibited degranulation of mast cells and membrane translocation of PKC epsilon	Dose dependent increase of thermal hyperalgesia and mechanical allodynia thresholds	[41]
Icariin	Paclitaxel induced	Male Sprague Dawley rats	IG/25, 50, 100 mg/kg.	Suppressed GFAP and astrocyte production of TNF-α, IL-1b, and IL-6.	Alleviated mechanical allodynia (100 mg/kg in the long term) and spinal neuroinflammation.	[42]

**Table 7:** Previous studies investigating the mechanisms by which anticancer drugs exert their effects.

Mechanism of CIPNP	Neuropathic Pain Model	Mode of Administration/ Concentration	Animal	Reference
Increased sphingosine metabolism and consequently increased ceramide, DH-S1P, and SIP. Increased TNF-α and IL-1β in blood plasma	Bortezomib induced	Intraperitoneal/total 1 mg/kg over 5 consecutive days (0.2 mg/kg per day) and Intraperitoneal/0.4 mg/kg every other day 3 times a week for 4 weeks	Male Sprague Dawley rats and GFAP-Cre breeder mice	[67]
Upregulated TREM 2 ligand and thus increased TREM2/DAP12 complex signaling, leading to the activation of microglial cells	Cisplatin induced	Intraperitoneal/ 23mg/kg spread over 2 rounds of 5 consecutive days with a 5day break	Adult male mice	[68]
Increase in the astrocyte-specific gap junctional protein CX43 in the spinal cord, leading to enhanced astrocyte activation.	Oxaliplatin induced	Intraperitoneal/4 injections of 2 mg/kg each, every other day	male Sprague-Dawley rats	[65]
Downregulation of GLAST and GLT-1 in the spinal cord dorsal horn led to excessive activation of postsynaptic AMPA and NMDA receptors	Paclitaxel induced	Intraperitoneal/1 mg/kg per day for 4 consecutive days Intraperitoneal/1.0 mg/kg on 4 alternate days, total 4 mg/kgIntraperitoneal/2 mg/kg every other day for total 4 injections	male Sprague–Dawley ratsadult male Sprague–Dawley rats Adult male Sprague-Dawley rats	[66,74,75]
TLR4, through MYD 88 and TRIF, plays an integral role in nociceptive signaling.	Cisplatin induced	Intraperitoneal/ Six injections of 2.3 mg/kg given every other day	Wild type C57BL/6 mice; Tlr3–/–, Tlr4–/–, and Myd88–/– mice	[64]
TLR4 signaling in the spinal cord dorsal horn and DRG induces and maintains CIPN	Paclitaxel induced	Intraperitoneal/4 injections of 2 mg/kg administered every other day	Male Sprague-Dawley rats	[60]

**Table 8:** Previous studies investigating the effects of flavonoids on CIPN models.

Flavonoid	Neuropathic Pain Model	Animal	Mode of Administration/Concentration of Flavonoid	Mechanism-based Intervention	Effect on Neuropathy	Reference
Icariin	Paclitaxel induced	Male Sprague Dawley rats	IG/25, 50, 100 mg/kg	Suppressed GFAP and astrocyte production of TNF-α, IL-1β, and IL-6.	Alleviated mechanical allodynia (100 mg/kg in the long term) and spinal neuroinflammation.	[42]
Astragali radix	Oxaliplatin-induced neurotoxicity	Male Sprague Dawley rats	50% hydroalcoholic extracts of Astragali radix	Numerical reduction of astrocytes within the dorsal horns (demonstrated by GFAP immunohistochemistry)	Reduction of Oxaliplatin-induced molecular and morphometric alterations in peripheral nerve and dorsal-root ganglia. Decrease in the activation of microglia and astrocytes	[73]

**Table 9:** Previous studies investigating the mechanisms by which anticancer drugs exert their effects.

Mechanism of CIPNP	Neuropathic Pain Model	Mode of Administration/Concentration	Animal	Reference
Downregulation of MnSOD through post translational nitration by peroxynitrite.	Paclitaxel, oxaliplatin, bortezomib	Intraperitoneal; Paclitaxel: 4 doses delivered on alternate days, cumulative dose of 8 mg/kg Oxaliplatin: 10 mg/kg delivered over 5 consecutive days Bortezomib: 1 mg/kg delivered over 5 consecutive days	Male Sprague Dawley rats	[91]
Formation of DNA adducts	Cisplatin	2 microgram/ml for 48 h (in vitro)	Harlan–Sprague–Dawley rats and wild type background mice (C57BL/6J)	[85]
Increased permeability of mPTP	Paclitaxel	Intraperitoneal/ 1 ml/kg on 4 alternate days (in vitro and in vivo)	Adult male Sprague–Dawley rats	[92]
Increased LPS-induced iNOS expression	Oxaliplatin	i.v./nine injections of 1 mg/kg each given twice a week (in vitro and in vivo)	male Swiss mice	[56]

**Table 10:** Previous studies investigating the effects of flavonoids on CIPN models.

Flavonoid	Neuropathic Pain Model	Animal	Mode of Administration/Concentration of Flavonoid	Mechanism Based Intervention	Effect on Neuropathy	Reference
Genistein	Chronic constriction sciatic nerve injury	C57BL/6J male mice	Subcutaneously/once a day for 11 days at doses of 1,3,7.5,15,30 mg/kg (in vitro and in vivo)	Restoration of mitochondrial GPX levels, and reduction of LPS-induced iNOS production	Reversal of mechanical allodynia and thermal hyperalgesia in a time and dose-dependent manner	[81]
Morin	Chronic constriction injury	Male Sprague-Dawley rats	Oral/30 mg/kg for 14 days (in vitro and in vivo)	Reduced PARP overactivation and nitrite levels. Restored ATP and glutathione levels; repaired DNA damage	Reversed mechanical, chemical, and thermal hyperalgesia	[78]
Isoorientin	Chronic constriction injury	Adult, male specific pathogen free mice from ICR	Intragastric/7.5, 15 or 30 mg/kg per day	Ameliorated axonal swelling; prevented demyelination	Reduced hyperalgesia and allodynia	[80]
Quercetin and rutin	Oxaliplatin induced	male Swiss mice	i.v./nine injections of rutin (25, 50, and 100 mg/kg) or quercetin (25, 50, and 100 mg/kg) given twice a week (in vitro and in vivo)	Decreased LPS-induced iNOS expression	Inhibition of thermal and mechanical hyperalgesia	[56]
GSPE	Chronic constriction injury	Wistar rats of either sex	Oral/100 and 200 mg/kg for 14 days (in vitro and in vivo)	Increased SOD and GSH	Attenuation of thermal hyperalgesia and mechanical allodynia	[79]
Silibinin	Oxaliplatin	Rat model of painful oxaliplatin-induced neuropathy	Silibinin (100 mg/kg), administered once a day, starting from the first day of oxaliplatin injection until the 20th	Prevention of oxidative damage	Antineuropathic effects	[90]

## Abbreviations and Symbols

GSPE	Grape Seed Proanthocyanidins
CCI	Chronic Constriction Injury
CIPN	Chemotherapy Induced Peripheral Neuropathy
SNI	Sciatic Nerve Injury
SNL	Sciatic Nerve Ligation
PNL	Partial Nerve Ligation
LPS	Lipopolysaccharide
iNOS	Inducible Nitric Oxide Synthase
MnSOD	Manganese Superoxide Dismutase
mPTP	Mitochondrial Permeability Transition Pore
GAPDH	Glyceraldehyde 3 phosphate Dehydrogenase
GSH	Glutathione
GPX	Glutathione Peroxidase
TLR	Toll Like Receptor
DRG	Dorsal Root Ganglion
MAP	Mitogen Activated Protein
GFAP	Glial Fibrillary Acidic Protein
S1P	Sphingosine-1-phosphate
HPETE	5-hydroperoxyeicosatetraenoic acid
VGCC	Voltage Gated Calcium Channels
SGC	Satellite Glial Cells
ICW	Intracellular Calcium Waves
ROS	Reactive Oxygen Species
RNS	Reactive Nitrogen Species
RCS	Reactive Carbon Species
DPPH	2,2-diphenyl-1-picrylhydrazyl
SIRT1	Sirtuin 1
NT	Neurotransmitter
PAR	Protease Activated Receptor
NGF	Nerve Growth factor
LKB-1	serine–threonine liver kinase B1
